# Serological and Molecular Diagnosis of *Toxoplasma gondii* Infections in Thalassemia Patients

**Published:** 2019

**Authors:** Hooman HANIFEHPOUR, Seyed Kamal SAMSAM SHARIAT, Mohammad Saleh GHAFARI, Farnaz KHEIRANDISH, Vafa SABER, Shirzad FALLAHI

**Affiliations:** 1. Research Committee, Shahrekord University of Medical Sciences, Shahrekord, Iran; 2. Department of Medical Parasitology and Mycology, School of Medicine, Shahrekord University of Medical Sciences, Shahrekord, Iran; 3. Razi Herbal Medicines Research Center, Lorestan University of Medical Sciences, Khorramabad, Iran; 4. Department of Medical Parasitology and Mycology, School of Medicine, Lorestan University of Medical Sciences, Khorramabad, Iran; 5. Department of Medical Parasitology and Mycology, School of Medicine, Shahid Beheshti University of Medical Sciences, Tehran, Iran

**Keywords:** Thalassemia major, *Toxoplasma gondii*, Serology, Loop-mediated isothermal amplification

## Abstract

**Background:**

This study aimed to the serological and molecular diagnosis of *Toxoplasma gondii* infections and related risk factors in patients with thalassemia major and healthy controls.

**Methods:**

This case-control study was performed in Shahrekord University of Medical Sciences, Shahrekord, west of Iran from Jan 2014 to Jan 2015. Overall, 235 patients with thalassemia major and 235 healthy controls were enrolled. Assessment of anti-*Toxoplasma* antibodies in sera samples was performed using commercial ELISA kits. In order to the molecular investigate of *T. gondii* in blood samples, a relatively new molecular assay, LAMP technique based on *Toxoplasma* SAG1 gene was conducted for the first time. The specificity of LAMP outer primers for the *T. gondii* detection was confirmed by sequencing the purified PCR product.

**Results:**

51.9% of thalassemia patients and 34.8% of healthy controls were positive for anti-*Toxoplasma* IgG antibodies, which the difference was statistically significant (*P*<0.01). In terms of anti-*Toxoplasma* IgM antibody, 3.4% of thalassemia patients and 2.1% of healthy individuals were positive, which the difference was not statistically significant (*P*=1). Based on SAG1-LAMP, 9.78% of the thalassemia patients and 5.95% of healthy controls were positive for *T. gondii* DNA, which the difference was not statistically significant (*P*≤0.230).

**Conclusion:**

Thalassemia patients, probably due to repeated blood transfusion and consequently, immune deficiency, are at risk of transmitting *Toxoplasma* infection more than healthy people. Therefore, screening of *Toxoplasma* infection in blood transfusion centers may be effective in the prevention of toxoplasmosis in these patients.

## Introduction

Toxoplasmosis is one of the most common parasitic infections of human and animals caused by a protozoan of the *Apicomplexa* phylum, called *Toxoplasma gondii* (*T. gondii*). This parasite has a considerable dispersion throughout the world (1, 2). As an opportunistic parasite, *T. gondii* can be transmitted through blood transfusion and can be dangerous in immune-compromised people and pregnant women (2, 3). In people with an impaired immune system, patients with various malignancies and collagen vascular disease, reactivation of parasites can cause widespread inflammation and systemic involvement of many organs, especially the central nervous system, heart, and lungs (3). As a result of severe infection and widespread inflammation in the mentioned organs, encephalomyelitis, myocarditis, and pneumonia can occur and consequently in the absence of definitive early diagnosis and effective treatment can lead to death (2–4). In patients at risk, the accurate and timely diagnosis of disease is very helpful in controlling and treating the disease. Serological, biological, histological, and molecular methods or some combination of these assays are used to diagnosis the infection in humans (3, 5).

Thalassemia, also called Mediterranean anemia, is a form of inherited autosomal recessive blood disorder characterized by abnormal formation of hemoglobin (5). Immune status in patients with thalassemia has been investigated by studying cellular and humoral immune system (6). Due to a basic defect in the host defense, thalassemia has an increased risk for serious opportunistic infections such as toxoplasmosis and this may be associated with the chronic immune stimulation by repeated blood transfusions, iron overload, splenectomy and immune deficiency (7, 8). Precise early detection and consequently, effective treatment of toxoplasmosis as a dangerous opportunistic infection in thalassemia patients, is very useful in controlling and treating the disease.

Loop-mediated isothermal amplification (LAMP) technique, a relatively new DNA amplification technique, is an isothermal nucleic acid amplification technique which due to its simplicity, ruggedness, high sensitivity and specificity and low cost could provide major advantages (9, 10). This method can amplify a few copies of targeted DNA to 10^9^ in less than an hour (4, 10). The LAMP has already been successfully used for rapid detection of human infectious agents such as viruses, bacteria, parasites, and fungi (11).

The aim of the present study was to primary serologic screening and then sensitive and specific molecular detection of *T. gondii* infection using a SAG1-LAMP technique in patients with thalassemia major and healthy controls.

## Methods

### Design, subjects, settings, and inclusion

This case-control study was performed in Shahrekord University of Medical Sciences, Shahrekord, west of Iran from Jan 2014 to Jan 2015. The study population consisted of 235 thalassemia major patients referred to Thalassemia Clinic of Hajar Hospital, Shahrekord, Iran as the case group and 235 healthy subjects in the same age and sex distribution as the control group. Infection of patients with thalassemia major was confirmed according to the patient's history, medical records, blood tests and hemoglobin electrophoresis. In the control group, healthy individuals referred to healthcare centers in Shahrekord for the annual checkup were also selected after informing them about the aims of the study. Demographic/clinical data and informed written consent were obtained from all of the participants. Clinical and laboratory evaluation of controls was performed in the same way as for the cases.

### Identification of anti-Toxoplasma specific antibodies

After collecting 5 ml of whole blood samples from all participants, the samples were transferred to a Medical Research Laboratory in Parasitology Department of Shahrekord University with respect to cold chain conditions. All hyperlipidemic or hemolysis sera were excluded or replaced with suitable alternative samples. After separation of serum by centrifugation, the sera samples were kept at −20 °C until examination. To search for specific anti-*Toxoplasma* IgM and IgG antibodies in the serum samples, the ELISA kits (*Toxoplasma* IgG/IgM ELISA kit, *ACON* Laboratories, Inc., USA) which have sensitivity and specificity of more than 95% were used. The experimental procedures were carried out according to the kit manufacturer's protocol. Finally, absorbance was measured by an ELISA reader (Stat Fax 2100/ UK) at 450 nm.

### Buffy coat isolation and DNA extraction

In order to the optimal purification of buffy coat layer from blood samples, the white blood cells (WBCs) isolating solution was used (9). The DNA was extracted from buffy coat specimens and the mouse harvested *T. gondii* tachyzoites using the DNG-plus kit (Sina gene Co., Iran) based on the kit protocol. The light absorption was measured at a wavelength of 260/280 nm using NanoDrop spectrophotometer (ND-2000, Thermo Scientific/ USA).

### LAMP reaction

In order to carry out the LAMP on extracted DNA from blood samples of thalassemia major patients and healthy control group, the primers presented for the SAG1 gene of *T. gondii* (GenBank accession no. X14080) were used (2). The reaction mixture was used in a final volume of 25 μl, containing 1.4 mM dNTP, 0.8 M betaine, 8 U *Bst* DNA polymer-ase (New England Biolabs), 1X Buffer enzyme [Tris-HCL (20 mM), KCL (10 mM), MgSO_4_ (9 mM), (NH_4_)_2_SO_4_ (10 mM)], 5 pmol each of F3 and B3 primers, 40 pmol each of FIP and BIP primers, 6.5 μl DDW water, 1 μl of template DNA and 8 mM MgSO_4_. The content of the microtubes was thoroughly mixed by quick spin and put into the thermal cycler for DNA amplification at 65 °C for 60 min. In addition to the thermal cycler, water bath, and heater blocks were also used for DNA amplification. After completion of the LAMP reaction, 1 μl of 0.1% SYBR Green I was added to each tube and then the tubes were assessed under natural and UV light with a wavelength of 312 nm. Positive tubes were green while the negative tubes remained to pale orange color.

### Statistical analysis

Statistical analyses were performed using SPSS statistical software (version 20.0; Chicago, IL, U.S.A). All data were presented as the mean ± SD. To compare qualitative and quantitative variables, Chi-square and independent sample T-tests were used respectively. *P-*values less than 0.05 were considered statistically significant and 95% confidence intervals were reported.

### Ethical approval

The study protocol conforms to the ethical guidelines of the 1975 Declaration of Helsinki as reflected in a priori approval by the institution’s Ethics in Research Committee (Ref. No. 200/13/2015).

## Results

### Sociodemographic characteristics of participants

Overall, 470 participants (235 patients with thalassemia major and 235 healthy control subjects) participated in this study. The age range of thalassemia major patients was 10–80 yr with a mean age 48.6±16.58 yr and in the control group ranged from 10–78 yr with an average age of 48.1±16.81 yr. T-test showed no difference in the age of the two groups (*P*=0.95). Regarding gender, the thalassemia patients included 106 men (45.1%) and 129 women (54.8%) and the control group consisted of 109 males (46.3%) and 126 women (53.6%), which chi-square test showed no difference in terms of gender in the two groups (*P*=0.851).

### Seroprevalence of anti-Toxoplasma IgM and IgG antibodies

Out of 235 patients with thalassemia major, 122 patients (51.9%) were positive for anti-*Toxoplasma* IgG antibodies as well as 82 of 235 healthy individuals (34.8%) were positive for this antibody, therefore a significant difference was observed between the two groups (*P*<0.01). Regarding anti-*Toxoplasma* IgM antibodies, 3.4% of patients with thalassemia and 2.1% of healthy individuals were positive for this antibody (Table 1), therefore a statistically significant difference was not observed between the two groups (*P*=1).

**Table 1:** Demographic characteristics of thalassemia patients and healthy control group according to the *T. gondii* seropositivity

***Variable***	***Status***	***T^†^ Positive N (%)***	***P-value***	***C^‡^ Positive N (%)***	***P-value***
Age(yr)	10–19	23 (46.2)		16 (65.2)	
	20–29	16 (27.8)	0.001	6 (38.7)	
	30–39	18 (37.5)		8 (19.1)	0.001
	40–49	11 (36.9)		18 (25.7)	
	50–59	19(31.2)		13 (13.6)	
	>60	35 (52.6)		21 (65.3)	
	Total	122 (51.9)		82 (34.8)	
Sex	Male	43 (40.5)		39 (35.7)	0.001
	Female	79 (61.2)	0.001	43 (34.1)	
	Total	122 (51.9)		82 (34.8)	
Place of residence	Urban	63 (63.6)	0.871	71 (53.2)	0.754
	Rural	59 (56.4)		11 (45.2)	
	Total	122 (51.9)		82 (34.8)	
Contact with cat	Touch	74 (61.3)		56 (36.7)	
	Non-touch	48 (52.8)	0.001	26 (41.5)	0.693
	Total	122 (51.9)		82(34.8)	
	Illiterate	38 (57.3)	0.584	25 (59.2)	0.974
Education	Elementary	26 (47.1)		19 (32.1)	
	Guidance	17 (39.6)		25 (47.3)	
	High School	23 (46.2)		8 (24.1)	
	University	18 (28.9)		5 (31.1)	
	Total	122 (51.9)		82 (34.8)	
Job	Housekeeper	27 (46.5)	0.002	33 (52.4)	
	Employee	17 (29.8)		15 (13.9)	0.241
	Farmer	25 (36.7)		12 (19.4)	
	Rancher	53 (61.9)		22 (33.5)	
	Total	122 (51.9)		82 (34.8)	
Meat diet	Red meat	65 (47.2)	0.746	48 (56.2)	0.344
	White meat	57 (32.4)		34 (26.4)	
	Total	122 (51.9)		82 (34.8)	
Vegetable diet	Continuous	59 (37.4)	0.364	43 (48.2)	0.714
	Periodic	63 (42.1)		39 (26.5)	
	Total	122 (51.9)		82 (34.8)	
Meat cooking status	Cooked	41 (38.7)	0.001	29 (32.8)	0.124
	Undercooked	81 (64.3)		53 (41.5)	
	Total	122 (51.9)		82 (34.8)	

† Thalassemia patients,

‡ Control group

There is a statistically significant difference regarding the serum prevalence of *Toxoplasma-*specific IgG antibodies for age and sex variables in each group, as well as, the differences were also significant regarding contact with cats, job, and consumption of meat. Therefore, in terms of age, the highest prevalence was seen in the age group of 10–19 yr and above 60 yr and the prevalence was increased with age (*P*=0.001). With regard to gender, the prevalence was higher in men than women and a significant difference was also observed in both groups (*P*=0.001). The prevalence was higher in people who had contact with cats than the people who had no close contact with cats (*P*). Moreover, regarding the job variable, the prevalence of *Toxoplasma* infection was higher in housewives and butchers (*P*=0.002). The prevalence of *Toxoplasma* infection was also higher in people who consumed undercooked meat than those who consumed fully cooked meat (*P*=0.001) (Table 1). Results of the logistic regression model showed that in the presence of all the variables, age and sex are the most important factors in the prevalence of *Toxoplasma* infection in patients with thalassemia major (*P*=0.001). According to the odds ratio values and other variables values remaining constant in the statistical model, by addition of 1 year to the age, the risk of infection is 1.051 times greater. Moreover, considering the significance of gender, the chance of getting infected by *Toxoplasma* in women rises 1.011 times.

### LAMP reaction

**Fig. 1:**
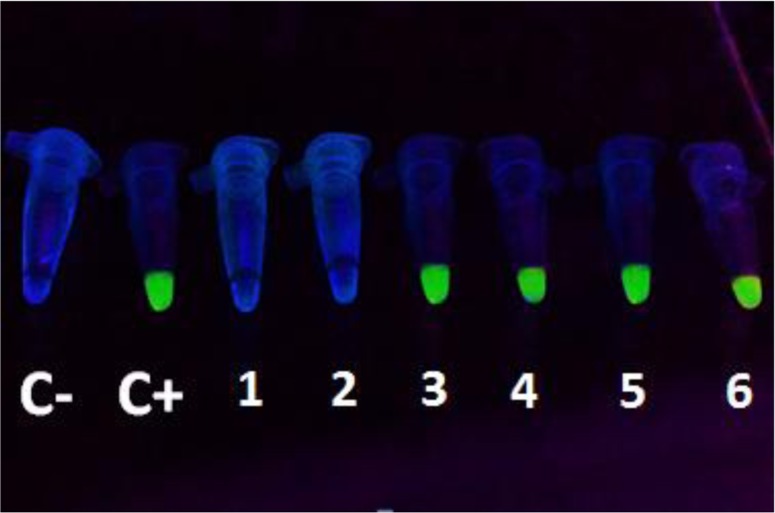
Analysis results of SAG1-LAMP on blood samples from thalassemia patients using SYBR Green I DNA stain under UV light. C-, negative control; C+, positive control; tubes 1–6, randomly selected samples from patients

**Fig. 2:**
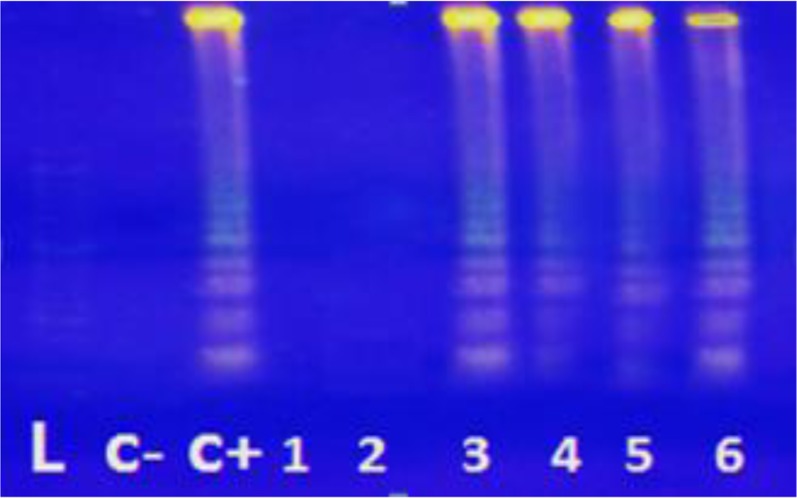
Analysis results of SAG1-LAMP of blood samples from thalassemia patients on 1.5% agarose gel stained with SYBR Safe DNA gel stain. L, 100 bp molecular weight marker; C-, negative control; C+, positive control; lanes 1–6, randomly selected samples from patients

Results of LAMP technique based on SAG1 gene of *Toxoplasma* showed that 23 samples (9.78%) in the major thalassemia patients group (Figs. 1 and 2) and 14 samples (5.95%) in the control group were positive for *T. gondii* DNA. This means that of 105 IgM−/IgG−, 8 IgM+/IgG+ and 122 IgM−/IgG+ samples in the case group, 5, 8 and 10 samples were positive using the SAG1-LAMP technique, respectively. Moreover, out of 148 IgM−/IgG−, 5 IgM+/IgG+ and 82 IgM−/IgG+ samples in the control group, 3, 5 and 6 samples were positive by SAG1-LAMP technique, respectively (Table 2).

**Table 2:** The results of SAG1-LAMP based on serologic status of thalassemia patients and healthy control group for *T. gondii*

***Studied groups***		***T N (%)***	***C N (%)***	***Total N (%)***	***P-value***
ELISA N (%)	IgG− / IgM−	105 (44.68)	148 (62.97)	253 (53.82)	> 0.05
	IgG+ / IgM+	8 (3.40)	5 (2.12)	13 (2.76)	1
	IgG+ / IgM−	122 (51.91)	82 (34.89)	204 (43.40)	<0.01
Total		235 (100)	235 (100)	470 (100)	
SAG1-LAMP Positive N (%)	IgG− / IgM−	5 (2.12)	3 (1.27)	8 (1.70)	
	IgG+ / IgM+	8 (3.40)	5 (2.12)	13 (2.76)	<0.23
	IgG+ / IgM−	10 (4.25)	6 (2.55)	16 (3.40)	
Total		23 (9.78)	14 (5.95)	37 (7.87)	

Based on the results of SAG1-LAMP, there was not a statistically significant difference in terms of age and sex in both groups so that the prevalence of *Toxoplasma* infection was higher in people over 60 yr and in women. As well as, for other parameters such as contact with cats, job, consumption of raw or under-cooked meat, place of residence, the level of education and vegetable consumption, there was no statistically significant difference.

## Discussion

For a long time, toxoplasmosis has been known as an opportunistic infection in the immunocompromised patients. It is also known as the third leading cause of death in AIDS patients after *Pneumocystis* and *Cryptosporidium*, which makes it an important issue (12–14). In this study, 55.31% of patients with thalassemia major and 37.02% of healthy individuals were positive for anti-*Toxoplasma* antibodies. The higher anti-*Toxoplasma* IgM and IgG antibodies titers in patients with thalassemia major compared with the control group may be due to that Thalassemic patients are more likely to be at risk for *Toxoplasma* infection than healthy individuals due to repeated blood transfusions. The results of this study were in contradiction with those in patients with thalassemia major (15) 19.4%, in neoplasia patients 63% (16), in patients with epilepsy 67.1% (17) and in hemodialysis patients 80% (18). On the other hand, the results were in line with those in cancer patients 45.2% (19) and in diabetic patients 52.6% (20). In this study, the titer of anti-*Toxoplasma* IgM antibody in patients with thalassemia major (3.4%), was almost compatible with the results of study in immunodeficiency and hemodialysis patients 2.3%, (21) and contradicted in neoplasia patients 6.5% (16), in cancer patients 10.3% (19), in blood donors 0.28% (22), and with those of (23) in kidney disease patients, 6.7%. Up to now, the reason for the differences in the results are not fully understood, but various factors such as environmental conditions, cultural habits, foods, and safety level of the people against this parasite, are the factors that can effect on the level of infection (12).

The prevalence of *Toxoplasma* infection increases with age so that in the fourth decade of life, the prevalence of infection reaches 90% (24). The results of present study showed a statistically significant difference in terms of the variable of age in both groups (*P*<0.05). These results conflict with some studies around the world (25, 26); but it was in line with another study (19, 20, 27). In this research, regarding gender, a significant difference was seen in both groups, that the prevalence of IgG antibodies was higher in women. Maybe because women are more susceptible to infection with *T. gondii* (while cleaning meat and vegetables at home) than men. This result is incompatible with the results of studies (18, 27) and in line with the results of (19,21,26) studies. Occupation and its association with prevalence of *Toxoplasma* infection have been addressed in some studies (28–31). In the present study, a statistically significant difference was seen in the case (patients with thalassemia major) and control (healthy individuals) group, so, the prevalence of *Toxoplasma* infection was higher in cases who were working at a slaughterhouse or as butchers (*P*<0.05). These people may have been more at risk for this infection because of their contact with fresh meat. In present research the seroprevalence of IgG antibodies in both groups was higher in individuals that had contact with cats than those who did not have any close contact with this animal and this difference was significant for both groups (*P*<0.05). The prevalence of toxoplasmosis is higher in people who work in slaughterhouses and with low literacy level than the others (32–36). People who are less educated are more exposed to this infection due to the lack of awareness regarding the ways of transmission and lack of personal hygiene. In the present study, the highest incidence of infection in both groups was for those who ate undercooked meat, and a statistically significant difference was observed for the case group.

Regarding the results of SAG1-LAMP technique, the prevalence of *T. gondii* infection in people over 60 yr and in women was higher than the others that this is probably due to a higher exposure to parasite, direct contact with cat and/or consumption of contaminated meat by tissue cyst, that there was no statistically significant difference (*P*>0.05). Since the *Toxoplasma* DNA was found using SAG1-LAMP in blood samples from 5 patients with thalassemia major and 3 subjects in the control group who’s their serology test result was negative, it is likely that in these people the *Toxoplasma* infection occurred recently and sampling was done before increasing the serum antibody titer. In this study, *T. gondii* DNA was found in blood samples from 10 patients with thalassemia major and 6 people in the control group who’s their serum test results were negative for the presence of IgM antibody, but positive for IgG antibodies. Since immune deficiency can cause reactivation of chronic and latent *Toxoplasma* infections, the existence of parasite DNA in blood samples may prove that the infection recently converted from chronic to acute but the IgM antibody titers are not to the extent that is detectable in the serum, yet. In addition, SAG1-LAMP technique on blood samples from 8 patients with thalassemia major and 5 subjects in the control group who had the IgG and IgM antibodies in their serum, approved the existence of the parasite's DNA. Although, this finding may indicate the presence of acute *Toxoplasma* infection in individuals, but to prove this diagnosis it is needed to perform additional supplementary tests such as inoculation of the patient's blood to sensitive animals or to cell cultures such as MRC5 or Vero cell lines as well as conducting complementary serum tests like IgG avidity (1,12). Since the sensitivity and specificity of diagnostic LAMP technique are much higher than the conventional molecular diagnostic methods such as PCR or common serology tests, it can be used to identify acute toxoplasmosis in humans and especially in immunocompromised people, including thalassemia major patients.

## Conclusion

Thalassemia patients are more likely to be at risk for *Toxoplasma* infection than healthy individuals due to repeated blood transfusions. Therefore, screening blood for *Toxoplasma* infection in blood transfusion centers may be effective in preventing toxoplasmosis in these patients.
